# Twenty-year prospective cohort study of the association between a Japanese dietary pattern and incident dementia: the NILS-LSA project

**DOI:** 10.1007/s00394-023-03107-x

**Published:** 2023-02-17

**Authors:** Shu Zhang, Rei Otsuka, Yukiko Nishita, Chikako Tange, Makiko Tomida, Fujiko Ando, Hiroshi Shimokata, Hidenori Arai

**Affiliations:** 1 Department of Epidemiology of Aging, Center for Gerontology and Social Science, National Center for Geriatrics and Gerontology, 7-430 Morioka-cho, Obu, Aichi 474-8511 Japan; 2 Faculty of Health and Medical Sciences, Aichi Shukutoku University, Aichi, Japan; 3 Graduate School of Nutritional Sciences, Nagoya University of Arts and Sciences, Aichi, Japan; 4 National Center for Geriatrics and Gerontology, Aichi, Japan

**Keywords:** Japanese diet, Dementia, Cohort, Apoprotein E genotype

## Abstract

**Purpose:**

Evidence has suggested that adherence to a Japanese diet may be beneficial for health. However, its association with incident dementia remains unclear. The aim was to explore this association in older Japanese community-dwellers, taking apoprotein E genotype into consideration.

**Methods:**

A 20-year follow-up cohort study involving 1504 dementia-free older Japanese community-dwellers (aged 65–82 years) living in Aichi Prefecture, Japan, was conducted. Based on a previous study, a 9-component-weighted Japanese Diet Index (wJDI9) score (range − 1 to 12) was calculated using 3-day dietary record data and used as an indicator of adherence to a Japanese diet. Incident dementia was confirmed by the Long-term Care Insurance System certificate, and dementia events occurring within the first 5 years of follow-up were excluded. A multivariate-adjusted Cox proportional hazards model was used to calculate hazard ratios (HRs) and 95% confidence intervals (CIs) for incident dementia, and Laplace regression was used to estimate percentile differences (PDs) and 95% CIs (expressed in months) in age at incident dementia (i.e., dementia-free duration differences), according to tertiles (T1–T3) of wJDI9 scores.

**Results:**

The median (IQR) follow-up duration was 11.4 (7.8–15.1) years. During the follow-up period, 225 (15.0%) cases of incident dementia were identified. Because the smallest prevalence of incident dementia was 10.7% for the T3 group of wJDI9 scores, to avoid inaccurately estimating the dementia-free duration of participants in the T3 group, the 11th PDs in age at incident dementia between the T1 and T3 groups of wJDI9 scores were estimated. A higher wJDI9 score was associated with a lower risk of incident dementia and a longer dementia-free duration difference. The multivariate-adjusted HR (95% CI) and 11th PDs (95% CI) in age at incident dementia for participants in the T1 vs. T3 group were 1.00 (reference) vs. 0.58 (0.40, 0.86), and 0 (reference) vs. 36.7 (9.9, 63.4) months, respectively. Each 1-point increase of the wJDI9 score was associated with a 5% lower risk of incident dementia (*P* value = 0.033) and 3.9 (0.3, 7.6) additional months of dementia-free duration (*P* value = 0.035). No differences were seen in sex or smoking status (current smoker vs. non-current smoker) at baseline.

**Conclusion:**

These findings suggest that adherence to a Japanese diet defined by wJDI9 is associated with a lower risk of incident dementia in older Japanese community-dwellers, suggesting the benefit of the Japanese diet for dementia prevention.

**Supplementary Information:**

The online version contains supplementary material available at 10.1007/s00394-023-03107-x.

## Introduction

Dementia is a syndrome that affects memory, thinking, behavior, and ability to perform everyday activities, which poses an enormous burden on patients, their caregivers, and society [1]. In 2019, more than 6.5 million older Japanese (aged ≥ 65 years) needed long-term care services, 18% of which were caused by dementia [2]. Therefore, the identification and development of population- and individual-based prevention strategies aimed at reducing the incidence of dementia constitute an important health and economic priority.

A systematic review and meta-analysis showed that adherence to a healthy dietary pattern was associated with a lower risk of dementia [3]. The most well-known Mediterranean diet has been suggested to protect against cognitive decline and dementia [4]. However, it is not easy for Asians to adhere to the Mediterranean diet in terms of food culture. Compared to the Westernized diet in Japan today, the traditional Japanese diet reduces the risk of dementia [5]. Prospective cohort studies with follow-up for 5.7 years have reported that adherence to the traditional Japanese diet was associated with a lower risk of incident dementia [6, 7].

Nevertheless, to assess adherence to the traditional Japanese diet, previous studies used Japanese diet indices (the Japanese Diet Index, JDI and the 8-item Japanese Diet Index, JDI8; see Supplementary Table 1 for scoring details), which apply the same weight to all score components. This implies that all score components have the same impact on preventing dementia, which is unlikely to happen in most cases. Conceptually, assigning weights to some of the components is a better approach [8]. Our previous research on the nutritional characteristics of the traditional Japanese diet developed a 9-component-weighted Japanese Diet Index (wJDI9; see Supplementary Table 1 for scoring details), which assigns different weights to score components according to the strength of the correlation between score components and nutrient density (redefined as the percentage of daily actual intake of 11 nutrients relative to the Dietary Reference Intake for Japanese [9]) [10]. The study also observed that the nutrient density of wJDI9 is higher than that of JDI [10]. However, the selection of nutrients in that study did not focus on dementia prevention, and considering that the digestion, absorption, utilization, and interaction of food components and nutrients in the body is a complex process, whether wJDI9 also has a beneficial effect on preventing dementia is still unknown. Moreover, since the follow-up duration of previous studies was relatively short, there is a great need to explore the long-term preventive effects of the Japanese diet on dementia. In addition, given that apoprotein E (APOE) genotypes play a critical role in the pathogenesis of dementia, it will also be helpful to consider their influence.

Therefore, this study aimed to explore the association between adherence to wJDI9 and the risk of incident dementia in a long-term (up to 20 years) prospective cohort study of older Japanese community-dwellers, taking APOE genotype into consideration.

## Materials and methods

### Study cohort

Data from the present study were collected as part of the National Institute for Longevity Sciences-Longitudinal Study of Aging (NILS-LSA) project. The NILS-LSA is a Japanese population-based prospective cohort study of normal aging and age-related diseases. Participants were recruited via age- and sex-stratified random sampling from the neighborhoods of the institute, Obu City and Higashiura Town in Aichi Prefecture, Japan. The first-wave examination of the NILS-LSA was conducted from November 1997 to April 2000 and included 2267 participants (aged 40–79 years). These participants were followed-up every 2 years, and participants (aged 40–79 years) who were unable to participate in the follow-up survey were replaced by new, randomly recruited, age- and sex-matched participants. Participants aged 40 years were also newly recruited every year. Details of the NILS-LSA have been reported previously [11].

**Fig. 1:**
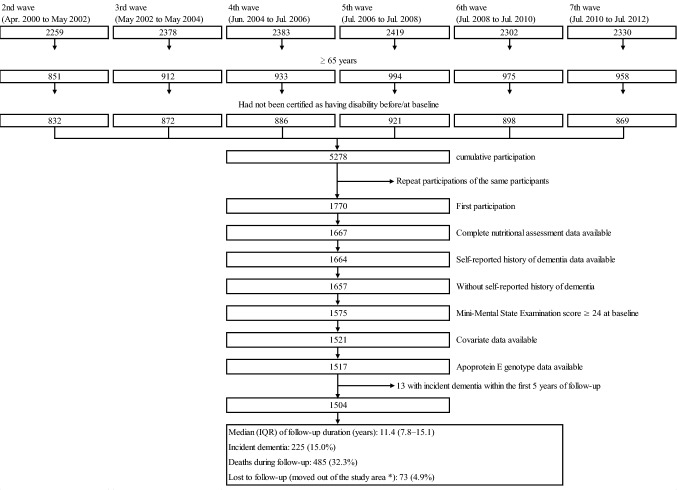
Flowchart of participants in the present study. *Without developing incident disability

Participants in this study were selected from the second (April 2000 to May 2002) to seventh (July 2010 to July 2012) waves of the NILS-LSA because the Long-term Care Insurance (LTCI) information was available since April 2000. In this study, for each participant, initial participation after reaching old age (≥ 65 years) was used as baseline (April 2000 to July 2012), and follow-up started from the date of baseline participation and ended on August 31, 2020. Of the 1,770 first participated older individuals who had not been certified as having disability (see definition below) before/at the baseline survey, 103 participants whose nutritional assessment data were incomplete at baseline, three whose self-reported history of dementia data were unavailable at baseline, seven who had a self-reported history of dementia, 82 whose Mini-Mental State Examination (MMSE) score was ≤ 23 points at baseline, 54 whose covariate data were incomplete at baseline, four whose APOE genotype data were unavailable, and 13 whose dementia event occurred within a follow-up of 5 years were excluded. Thus, 1504 Japanese older individuals (733 men and 771 women, aged 65–82 years) were analyzed in this study (Fig. 1).

### Dietary assessments

Dietary intakes were assessed using 3-day dietary records (3DRs). The dietary record was completed over 3 continuous days (2 weekdays and 1 weekend day), and most participants completed it at home and returned the records within 1 month. Food was weighed separately on a scale (1-kg kitchen scales; Sekisui Jushi, Tokyo, Japan) before being cooked or portion sizes estimated. Participants also recorded their diet using a disposable camera (27 shots; Fuji Film, Tokyo, Japan) by taking photos of meals before and after eating. Dietitians used these photos to complete missing data, and telephoned participants to resolve any discrepancies or obtain further information when necessary. Averages for 3-day nutrient and energy intakes were calculated according to the Standard Tables of Foods Composition 2010 in Japan and other sources [12].

### The 9-component-weighted Japanese dietary index

According to our previous study [10], we identified the wJDI9 by the following 9 components: “rice”, “fish and shellfish”, “green and yellow vegetables”, “seaweed”, “green tea”, “fruit”, “soybeans and soybean foods”, “mushrooms”, and “beef and pork (internal organs excluded)”. All components of the indices were classified based on the definitions used in the National Health and Nutrition Survey 2011 (Japan) [13]. By using the sex-specified median values of daily consumption for each food component as cut-off points, participants were assigned different scores according to their food consumption being below, or above and equal to, the corresponding median values. Last, the scores of all food items were added together to get a total value. The wJDI9 score ranged from − 1 to 12 (see Supplementary Table 1 for scoring details).

### Case (incident dementia) ascertainment and follow-up

The primary outcome was incident dementia, defined as disabling dementia consistent with the LTCI system standards used in Japan [14]. The LTCI is a mandatory national social insurance plan designed to assist disabled older individuals in carrying out activities of daily living [15, 16]. Everyone aged ≥ 40 years is required to pay premiums, and those who are aged ≥ 65 years are eligible to receive formal care services under a uniform disability certification standard. The procedure for disability certification consists of two parts: (1) using a questionnaire developed by the Ministry of Health, Labour and Welfare by a care manager to assess the degree of functional disability, and (2) referring to the Doctor’s Opinion Paper prepared by the attending physician [17]. Then, the administrative region government determines whether the applicant is eligible for LTCI benefits (certification) based on the above information.

According to the Levels of Independence Degree in Daily Living for Elderly with Dementia (LIDDLED) in the Doctor’s Opinion Paper, the independence of the applicant was classified into six ranks (0, I–IV, and M). In this study, a rank over or equal to II was considered to be an indicator of incident dementia for the LTCI system because individuals classified as rank II would need some kind of care/support due to having mild or moderate dementia [18]. A previous study showed that the LIDDLED is well correlated with the MMSE score (Spearman rank correlation coefficient = − 0.736) [19].

The person-years of follow-up for each participant were counted from the first participation date until the (1) date of incident dementia (LTCI certification), (2) date of non-dementia disability (LTCI certification), (3) date of death, date of moving out of Obu City and Higashiura Town, or (4) the end of the study period (August 31, 2020), whichever occurred first.

A data set that included information on LTCI certification, death, or emigration was obtained from Obu City and Higashiura Town. All data were transferred from the Obu City and Higashiura Town Government under an agreement related to Epidemiologic Research and Privacy Protection.

### Covariates

In the baseline survey, body mass index (kg/m^2^) was calculated as weight (in kilograms) divided by the square of height (in meters). Data on the history of disease (stroke, hypertension, dyslipidemia, and diabetes mellitus; yes or no, for each), smoking status (never, former, or current), education level (years; ≤ 9, 10–12, or ≥ 13), and marital status (married, or others) were collected by a self-administered questionnaire. The 24-h total physical activity was assessed by the metabolic equivalent of task (MET) score (METs*hr/day; continuous) obtained from participant interviews conducted by trained interviewers using a semiquantitative assessment [20]. Depressive symptoms were assessed by the self-administered Center for Epidemiologic Studies Depression (CES-D) Scale [21, 22] (CES-D score ≤ 15 or ≥ 16; representing normal status or the presence of relevant depressive symptoms, respectively). The MMSE [23] (Japanese version [24]) score was assessed by interviews with trained clinical psychologists or graduate students with a psychology major. Genomic DNA was extracted from peripheral blood lymphocytes using standard procedures. APOE genotypes were determined by polymerase chain reaction amplification [25]. Alcohol drinking (g/day), salt intake (g/day), and energy intake (kcal/day) were assessed through 3DRs.

### Ethics approval and consent to participate

The Committee on the Ethics of Human Research of the National Center for Geriatrics and Gerontology approved the study protocol (No. 899-6). Written, informed consent was obtained from all participants.

### Statistical analysis

The wJDI9 scores were divided into three groups (T1–T3) using tertiles of scores as the cut-off points. The wJDI9 score ranges in each group (T1–T3) ranged from − 1 to 4, 5 to 7, and 8 to 12 points, respectively. The median (IQR) value of the wJDI9 score was 5 (4–8) points.

A multivariate-adjusted Cox proportional hazards model was used to calculate hazard ratios (HRs) and 95% confidence intervals (CIs) for incident dementia according to the three groups of wJDI9 scores, using age as the time scale to account for left truncation and right censoring. Model 1 was adjusted for baseline information on sex, APOE genotype (APOE-ε4 carriers: 2/4, 3/4, 4/4, or APOE-ε4 noncarriers: 2/2, 2/3, 3/3), body mass index (kg/m^2^; < 18.5, 18.5 − < 25, or ≥ 25), history of disease (stroke, hypertension, dyslipidemia, and diabetes; yes or no, for each), and participation waves (categorical). Model 2 was adjusted for Model 1 plus baseline information on smoking status (never, former, or current), alcohol intake (g/day; continuous), total physical activity (METs*hr/day; continuous), education level (years; ≤ 9, 10–12, or ≥ 13), depressive symptoms (CES-D score; ≤ 15 or ≥ 16), marital status (married, or others), and energy intake (kcal/day; continuous). Model 3 was adjusted for Model 2 plus baseline information on the MMSE score (continuous). After applying the multivariate-adjusted Cox proportional hazards model, the proportional hazards assumption was tested by including a time-varying covariate (the interaction between the wJDI9 score and event time). Tests of interaction were performed by sex and smoking status (current smoker vs. non-current smoker).

In addition, since the percentage of incident dementia in the T3 group of wJDI9 was only 10.7% (the smallest percentage of all three groups), the 11th percentile differences (PDs) and 95% CIs (expressed in months) in age at incident dementia (i.e., dementia-free duration differences) according to the wJDI9 groups were calculated using a Laplace regression model [26, 27], adjusted by variables that were the same as those in Cox Model 3 plus age at baseline (years; 65–69, 70–74, 75–79, or ≥ 80).

Further, since prospective studies suggest detectable cognitive deficits may be detected as long as 10–12 years before dementia diagnosis [28, 29], to address possible reverse causality, a sensitivity analysis was conducted by excluding participants who developed dementia within the first 10 years of follow-up. Considering that participants with an MMSE score between 24 and 27 at baseline may have mild cognitive impairment, and thus their wJDI9 scores might be lower and they may be more likely to develop dementia during follow-up, the analysis was also repeated with only the 980 participants with MMSE scores ≥ 28 at baseline.

**Fig. 2:**
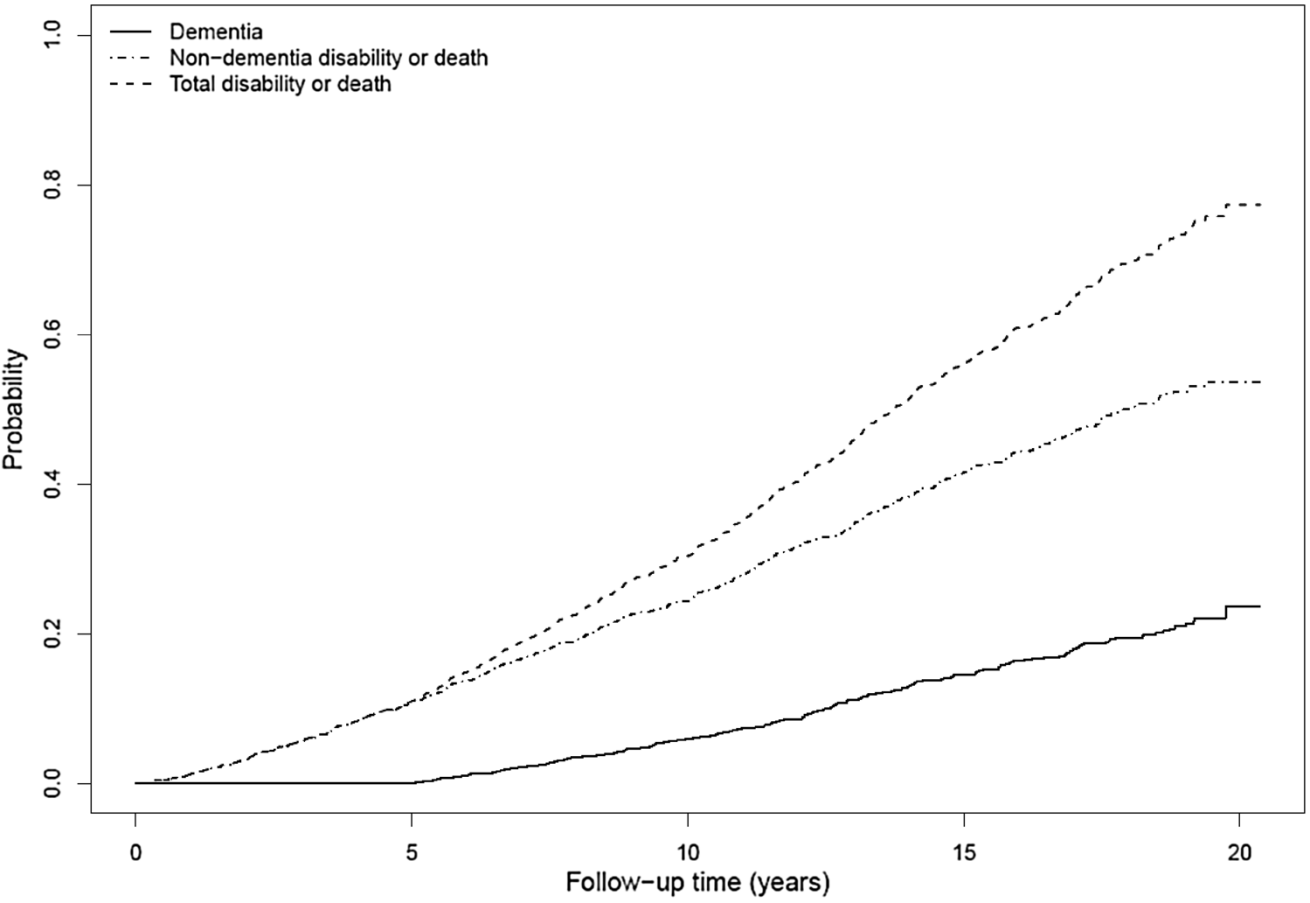
Cumulative incidence functions for the event of interest (dementia) and the competing event (non-dementia disability or death). Drawn by “*cmprsk”* package of R

Finally, since the percentage of death and non-dementia disability events during the follow-up period was not negligible (Fig. 2), a competing risk regression analysis was also performed using death and non-dementia disability as competitive events. The cumulative incidence function was modeled by defining the sub-distribution hazards, imposing the proportional hazards assumption on it [30], and adjusting for the same covariates in Model 3.

All statistical analyses described above were two-sided, accompanied by the exact *P* value. The Cox proportional hazards model was performed using Statistical Analysis System software version 9.3 (SAS Institute, Cary, NC, USA). Estimation performed by R version 4.0.5 (“*powerSurvEpi*” package) showed that, compared with the T1 group of wJDI9, the sample size and the number of dementia cases in the present study were sufficiently large to detect an HR of 0.584 for the T3 group, with a statistical power of 80% and a significance level of 5%. The Laplace regression model was performed using Stata/MP 16.1 provided by StataCorp LLC, TX.

## Results

### Baseline characteristics

Table 1 shows the baseline characteristics of the participants according to the wJDI9 tertile groups. Participants in the T3 (i.e., the highest tertile) group were more likely to have education of more than 13 years, to have higher intake of energy and salt, and to consume fish and shellfish, green and yellow vegetables, seaweed, green tea, soybeans and soybean foods, mushrooms, and fruit. Meanwhile, they were less likely to be current smokers and to consume rice, beef, and pork.

**Table 1:** Baseline characteristics (*n* = 1504)

	Tertiles of wJDI9 scores						*P* value^a^
	T1		T2		T3		
No. of participants	549		582		373		
wJDI9 score; mean (SD)	2.5	(1.4)	6.0	(0.8)	9.2	(1.1)	< 0.001
Age (years); mean (SD)	71.0	(4.8)	70.3	(4.6)	70.6	(4.7)	0.047
Women; %	51.9		50.9		50.9		0.929
APOE-ε4 carriers^b^; %	21.5		18.0		16.6		0.139
Mini-Mental State Examination score (points); mean (SD)	27.8	(1.7)	28.0	(1.6)	28.0	(1.6)	0.065
Body mass index (kg/m^2^); mean (SD)	23.0	(3.3)	23.1	(3.0)	22.7	(2.7)	0.153
Medical history (yes); %							
Stroke	5.1		8.4		5.1		0.037
Hypertension	40.1		41.1		40.5		0.943
Dyslipidemia	24.6		26.0		25.7		0.859
Diabetes mellitus	9.3		11.7		13.1		0.168
Current smoker; %	17.9		11.0		8.6		< 0.001
Alcohol (g/day); mean (SD)	7.9	(15.0)	7.9	(13.8)	8.8	(16.6)	0.618
Total physical activity (METs*hr/day); mean (SD)	34.3	(3.6)	34.1	(3.2)	34.3	(2.9)	0.519
Education level (years); %							
≤ 9	43.9		33.3		28.4		< 0.001
10−12	18.0		17.5		18.0		
≥ 13	38.1		49.1		53.6		
Married; %	77.6		80.8		83.9		0.058
Depressive symptoms^c^; %	12.6		11.7		9.7		0.389
Energy intakes (kcal/day); mean (SD)	1916.2	(379.0)	2028.3	(406.5)	2103.8	(404.4)	< 0.001
Salt intakes (g/day); mean (SD)	10.4	(2.5)	11.3	(2.7)	11.9	(2.6)	< 0.001
Food consumption (g/day); mean (SD)							
Rice	341.5	(149.0)	326.3	(141.4)	303.0	(125.7)	< 0.001
Fish and shellfish	85.0	(46.3)	101.3	(51.4)	113.4	(50.9)	< 0.001
Green and yellow vegetables	76.9	(41.6)	134.0	(69.0)	182.0	(70.3)	< 0.001
Seaweeds	13.4	(16.8)	18.4	(21.1)	26.1	(24.5)	< 0.001
Green tea	340.4	(322.3)	467.0	(381.5)	505.2	(375.2)	< 0.001
Beef and pork	40.8	(28.9)	38.2	(27.9)	33.6	(27.4)	< 0.001
Soybeans and soybean foods	46.9	(41.7)	75.2	(77.5)	95.7	(50.4)	< 0.001
Mushrooms	8.4	(10.8)	14.9	(17.4)	19.0	(16.3)	< 0.001
Fruit	113.3	(111.3)	164.4	(128.0)	222.6	(114.7)	< 0.001

*wJDI9* 9-component-weighted Japanese diet index

^a^For continuous variables, the general linear model was used; for categorical variables, the *χ*^2^ test was used

^b^APOE genotype was defined as APOE-ε4 carriers (2/4, 3/4, 4/4) and APOE-ε4 noncarriers (2/2, 2/3, 3/3)

^c^Defined by the Center for Epidemiologic Studies Depression Scale (CES-D) score ≥ 16

### wJDI9 and incident dementia

**Fig. 3:**
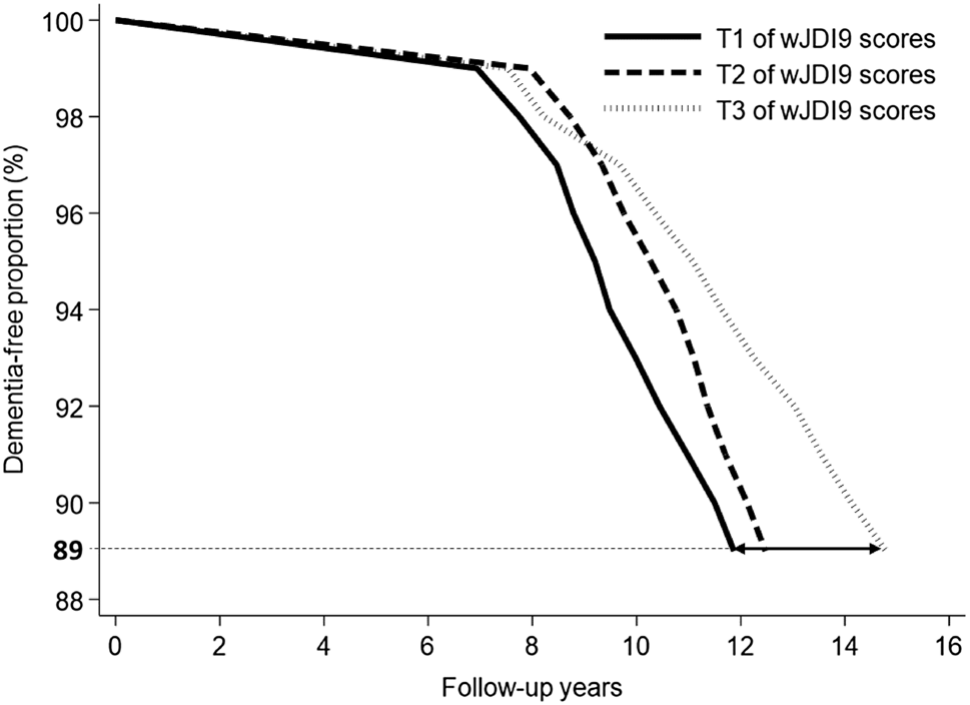
Dementia-free proportion (%) by follow-up years; drawn by Laplace regression and stratified by tertiles of the wJDI9 score at baseline. Adjusted for baseline information on age (years; continuous), sex, APOE genotype (APOE-ε4 carriers: 2/4, 3/4, 4/4, or APOE-ε4 noncarriers: 2/2, 2/3, 3/3), history of disease (stroke, hypertension, dyslipidemia, and diabetes mellitus; yes or no, for each), participation waves (categorical), smoking status (never, former, or current), alcohol intake (g/day; continuous), total physical activity (METs*hr/day; continuous), education level (years; ≤ 9, 10–12, or ≥ 13), depressive symptoms (CES-D score; ≤ 15 or ≥ 16), marital status (married, or others), energy intake (kcal/day; continuous), and MMSE score (continuous). Drawn by “*laplacereg*” and “*laplace_surv*” packages of Stata

The median (IQR) follow-up duration was 11.4 (7.8–15.1) years. After multivariate adjustment for potential confounders, a higher wJDI9 score was associated with a lower risk of incident dementia and a longer dementia-free duration difference (Table 2; Fig. 3). The multivariate-adjusted HR (95% CI) and 11th PD (95% CI) in age at incident dementia for participants in the T3 group were 0.58 (0.40, 0.86) (*P* value = 0.006) and 36.7 (9.9, 63.4) (*P* value = 0.007) months older than those in the T1 group. Each 1-point increase of the wJDI9 score was associated with a 5% (*P* value = 0.033) lower risk of incident dementia and 3.9 (0.3, 7.6) (*P* value = 0.035) additional months of follow-up duration without dementia. The proportional hazard assumption was not violated (*P* value for interaction between the wJDI9 score and the event time = 0.501;* P* value for interaction between the tertile groups of wJDI9 scores and event time = 0.645).

**Table 2:** HRs and 11th PDs with corresponding 95% CIs for incident dementia by wJDI9 score (wJDI9 scores were used as continuous and categorical variables)

	No. of participants	No. of incident dementia (%)	Person years	Model 1^a,c^				Model 2^a,d^				Model 3^a,e^				Model 4^b,f^			
				HR	95% CI		*P* value	HR	95% CI		*P*-value	HR	95% CI		*P* value	11th PDs (months)	95% CI		*P* value
Continuous wJDI9 scores	1504	225 (15.0)	16880.8	0.94	0.90	0.99	0.010	0.95	0.90	0.99	0.029	0.95	0.90	0.995	0.033	3.9	0.3	7.6	0.035
Tertiles of wJDI9 scores																			
T1	549	94 (17.1)	5992.8	1.00	Ref		–	1.00	Ref		–	1.00	Ref		–	0.0	Ref		–
T2	582	91 (15.6)	6505.3	0.96	0.72	1.29	0.786	0.97	0.72	1.31	0.857	0.97	0.72	1.31	0.848	7.9	− 7.6	23.4	0.316
T3	373	40 (10.7)	4382.7	0.55	0.38	0.79	0.002	0.58	0.39	0.85	0.005	0.58	0.40	0.86	0.006	36.7	9.9	63.4	0.007

*wJDI9* 9-component-weighted Japanese diet index, *HR* hazard ratio, *PD* percentile difference, *CI* confidence interval

^a^Analysis by Cox proportional hazards model

^b^Analysis by Laplace regression model

^c^Adjusted for baseline information on sex, APOE genotype (APOE-ε4 carriers: 2/4, 3/4, 4/4, or APOE-ε4 noncarriers: 2/2, 2/3, 3/3), body mass index (kg/m^2^; < 18.5, 18.5 − < 25, or ≥ 25), history of disease (stroke, hypertension, dyslipidemia, and diabetes mellitus; yes or no, for each), and participation waves (categorical)

^d^Adjusted for Model 1 + baseline information on smoking status (never, former, or current), alcohol intake (g/day; continuous), total physical activity (METs*hr/day; continuous), education level (years; ≤ 9, 10–12, or ≥ 13), depressive symptoms (CES-D score; ≤ 15 or ≥ 16), marital status (married, or others), and energy intake (kcal/day; continuous)

^e^Adjusted for Model 2 + baseline information on MMSE score (continuous)

^f^Adjusted for Model 3 + baseline information on age (years; 65–69, 70–74, 75–79, or ≥ 80)

The inverse association between wJDI9 scores and incident dementia did not differ with regard to sex (*P* for interaction = 0.363) or smoking status (current smoker vs. non-current smoker; *P* for interaction = 0.802).

### Sensitivity analysis

Even when participants who developed dementia within the first 10 years of follow-up were excluded from the analysis, or only the participants with MMSE scores ≥ 28 at baseline were included, the lower risk of incident dementia in the T3 group of wJDI9 still remained (Table 3).

**Table 3:** HRs (and 95% CIs) for incident dementia by wJDI9 score in sensitivity analysis (wJDI9 scores were used as continuous and categorical variables)^a^

	No. of participants	No. of incident dementia (%)	Person years	Multivariable adjusted model^b^			
				HR	95% CI		*P* value
Incident dementia within the first 10 years of follow-up were excluded							
Continuous wJDI9 scores	1419	140 (9.9)	16238.2	0.95	0.89	1.01	0.114
Tertiles of wJDI9 scores							
T1	516	57 (11.1)	5794.6	1.00	Ref		–
T2	550	58 (10.6)	6230.5	0.97	0.66	1.43	0.891
T3	353	25 (7.1)	4213.1	0.56	0.34	0.93	0.024
Only included participants with MMSE score ≥ 28 at baseline							
Continuous wJDI9 scores	980	132 (13.5)	11433.5	0.92	0.86	0.98	0.009
Tertiles of wJDI9 scores							
T1	342	60 (17.5)	3894.4	1.00	Ref		–
T2	265	33 (12.5)	3028.5	0.82	0.52	1.28	0.384
T3	373	39 (10.5)	4510.7	0.52	0.34	0.8	0.003

*wJDI9* 9-component-weighted Japanese diet index, *HR* hazard ratio, *CI* confidence interval, *MMSE* mini-mental state examination

^a^Analysis by Cox proportional hazards model

^b^Adjusted for baseline information on sex, APOE genotype (APOE-ε4 carriers: 2/4, 3/4, 4/4, or APOE-ε4 noncarriers: 2/2, 2/3, 3/3), body mass index (kg/m^2^; < 18.5, 18.5 − < 25, or ≥ 25), history of disease (stroke, hypertension, dyslipidemia, and diabetes mellitus; yes or no, for each), participation waves (categorical), smoking status (never, former, or current), alcohol intake (g/day; continuous), total physical activity (METs*hr/day; continuous), education level (years; ≤ 9, 10–12, or ≥ 13), depressive symptoms (CES-D score; ≤ 15 or ≥ 16), marital status (married, or others), energy intake (kcal/day; continuous), and MMSE score (continuous)

### Competing risk analysis

The total number (%) of events of interest (dementia) and competing events (non-dementia disability or death) and censored value were 225 (15.0%), 617 (41.0%), and 662 (44.0%), respectively. The results of the competing risk regression analysis are shown in Table 4. Again, compared to participants in the T1 group, those in the T3 group had a lower risk of incident dementia.

**Table 4:** HRs (and 95% CIs) for incident dementia by wJDI9 score (other disability or death was used as the competing event; wJDI9 scores were used as continuous and categorical variables)^a^

	No. of participants	Multivariable adjusted model^b^			
		HR	95% CI		*P* value
Continuous wJDI9 scores	1504	0.96	0.92	1.01	0.124
Tertiles of wJDI9 scores					
T1	549	1.00	Ref		–
T2	582	0.97	0.72	1.32	0.856
T3	373	0.62	0.42	0.92	0.017

*wJDI9* 9-component-weighted Japanese diet index, *HR* hazard ratio, *CI* confidence interval

^a^Analysis by competing-risk regression model

^b^Adjusted for baseline information on sex, APOE genotype (APOE-ε4 carriers: 2/4, 3/4, 4/4, or APOE-ε4 noncarriers: 2/2, 2/3, 3/3), body mass index (kg/m^2^; < 18.5, 18.5 − < 25, or ≥ 25), history of disease (stroke, hypertension, dyslipidemia, and diabetes mellitus; yes or no, for each), participation waves (categorical), smoking status (never, former, or current), alcohol intake (g/day; continuous), total physical activity (METs*hr/day; continuous), education level (years; ≤ 9, 10–12, or ≥ 13), depressive symptoms (CES-D score; ≤ 15 or ≥ 16), marital status (married, or others), energy intake (kcal/day; continuous), and MMSE score (continuous)

## Discussion

In this 20-year, population-based, cohort study, the Japanese dietary pattern defined by the wJDI9 score was associated with a lower risk of incident dementia and longer follow-up years without dementia. If we focus on the follow-up time, as of the time when the 11th percentile of participants in each group developed dementia, compared to individuals in the lowest tertile group of the wJDI9 score, individuals in the highest tertile group had a 36.7-month (approximately 3.1 years) longer follow-up time (i.e., dementia-free life span).. To the best of our knowledge, this is the first long-term cohort study to have investigated the association between the wJDI9 score and incident dementia.

### Comparison with previous studies

Previous studies suggested that adherence to similar Japanese dietary indices (JDI [6] and JDI8 [7]) was associated with a lower risk of incident dementia. However, in the present study population, an association was observed neither for JDI nor for JDI8 (Supplementary Table 2).

Compared to previous studies, the following reasons may have mainly contributed to the inconsistency in the findings. (1) The wJDI9 included different score components and score weights. Based on JDI, we added the fruit, soybeans and soybean foods, and mushrooms and excluded miso soup, pickles, and coffee in the index. In addition, green and yellow vegetables, fruit, and soybeans and soybean foods were given more weight than other food items, and rice was given a negative weight; (2) the follow-up time was much longer for this study (20 vs. 5.7 years in previous studies), and thus the present findings may show a long-term association; and (3) the MMSE and APOE genotype were taken into account, so that the present results may provide findings with fewer residual confounding factors.

### Mechanisms

Our previous validity study suggested that wJDI9 was correlated with a higher nutrient density score (a surrogate indicator of good nutrient intakes; simply put, high intakes of protein, fiber, Vitamin A, Vitamin C, Vitamin E, calcium, iron, potassium, magnesium, and low intakes of saturated fat and sodium) than JDI and a 12-component modified Japanese Diet Index (mJDI12) [10] (see Supplementary Table 1 for scoring details; mJDI12 was also not associated with incident dementia, Supplementary Table 2). Although the selection of the above nutrients did not focus on dementia prevention, high intakes of protein, Vitamin A, and Vitamin E, and low intakes of saturated fat and sodium have been linked to a lower risk of cognitive decline and dementia [31]. In addition, foods of wJDI9 associated with these nutrients are also high in nutrients and biochemical components that may prevent dementia, such as (−)-epigallocatechin-3-gallate in green tea [32], n-3 fatty acids in fish [31], and B vitamins in fish, mushrooms, legumes, fruit, and green and yellow vegetables [31]. Nevertheless, the present post hoc analysis found no association between single food components (except soybeans and soybean foods) and incident dementia (Supplementary Table 3), suggesting that focusing on dietary pattern (as an integration of nutrients and foods) may be more important in dementia prevention.

The high salt content of Japanese dietary pattern has been criticized to this day. We have noticed that adherence to a Japanese diet defined by wJDI9 is also accompanied by higher salt intake. Therefore, the salt intake in the post hoc analysis was adjusted to explore whether controlling for the salt intake in the Japanese diet would bring additional benefits to the prevention of dementia. Intriguingly, the results did not change substantially (Supplementary Table 4). In the present participants, the top 3 adherence foods of wJDI9 that were associated with salt intake were fish and shellfish, seaweed, and soybeans and soybean foods (data not shown). Because these foods also have beneficial effects on dementia prevention, therefore, they may offset the detrimental effects of concomitant salt intake.

### Strengths and limitations

Compared with previous studies, the present work had several strengths. First, the follow-up duration (up to 20 years) is the longest reported so far (previous up to 5.7 years). This made reverse causality less likely. Second, this study adjusted for many covariates, including APOE genotype and MMSE score. Third, the estimation of PD makes the results more intuitive and plays an important role in the popularization of knowledge for the general public and the formulation of public health policies.

Several limitations should also be noted. First, in order to ensure a sufficient sample size, only the baseline wJDI9 scores (first participation after reaching an age ≥ 65 years) were used in the analysis; participants may have changed their diet during follow-up. Second, because we used 3DRs to collect dietary information, although we asked participants to maintain their eating habits during the nutrition survey, participants may have consumed healthier foods than usual during the 3 days of the diet survey. However, we assume that this reporting bias may have significantly affected participants who usually eat unhealthily, resulting in the likelihood that participants with an unhealthy diet were assigned to the diet-healthy group, ultimately underestimating the beneficial effects of the Japanese diet defined by wJDI9. Third, despite adjustments for considerable covariates, residual confounding may have interfered with the findings. Fourth, the LTCI definition of dementia was used in this study, which differs from the clinical diagnosis of dementia. Fifth, since not all participants with disabilities would have applied for LTCI services during the follow-up, the possibility of detection bias could not be ruled out in the current study population. Last, 15% (= 266/1770) of older individuals were excluded for incomplete data. They were older, had lower baseline MMSE scores, worse health conditions and lifestyle behaviors, and higher incidence of dementia, suggesting that the present findings may only apply to healthier older community-dwellers. Further studies conducted in different settings are warranted.

## Conclusions

In conclusion, the results of this cohort study with follow-up of 20 years suggest that adherence to the Japanese diet defined by wJDI9 is associated with a lower risk of incident dementia in older Japanese community-dwellers. Compared to being treated, dementia seems to be a disorder that is easier to prevent or at least delay significantly. Exploring diets suitable for different populations and different cultures to prevent dementia is of utmost importance on a global scale.

## Supplementary Information

Below is the link to the electronic supplementary material.

Supplementary file1 (DOCX 30 KB)

## Data Availability

Data described in the manuscript and analytic code are available from the first or corresponding author based on a reasonable request.
