# Supplementary material for: Twenty-year prospective cohort study of the association between a Japanese dietary pattern and incident dementia: the NILS-LSA project
Source: Eur J Nutr. 2023 Feb 17;62(4):1719–29. doi: 10.1007/s00394-023-03107-x (PMC10195757; doi:10.1007/s00394-023-03107-x)
Supplement: Supplementary file 1 — Supplementary file1 (DOCX 30 KB) [file 394_2023_3107_MOESM1_ESM.docx]

| **Supplementary Table 1**. Scoring details of JDI ^a, b^, JDI8 ^c^, wJDI9 ^a^, and mJDI12 ^a^ | | | | | | | | | | | | |
| --- | --- | --- | --- | --- | --- | --- | --- | --- | --- | --- | --- | --- |
| Japanese diet indices | | Point (weight) of JDI | |  | Point (weight) of JDI8 | |  | Point (weight) of wJDI9 | |  | Point (weight) of mJDI12 | |
| Food components | | Daily intake (g/day) | |  | Daily intake (g/day) | |  | Daily intake (g/day) | |  | Daily intake (g/day) | |
|  |  | < gender-specific median | ≥ gender-specific median |  | < gender-specific median | ≥ gender-specific median |  | < gender-specific median | ≥ gender-specific median |  | < gender-specific median | ≥ gender-specific median |
| Rice | | 0 | 1 |  | 0 | 1 |  | 0 | -1 |  | 0 | 1 |
| Miso soup | | 0 | 1 |  | 0 | 1 |  | - | |  | 0 | 1 |
| Green and yellow vegetables | | 0 | 1 |  | 0 | 1 |  | 0 | 3 |  | 0 | 1 |
| Pickles | | 0 | 1 |  | 0 | 1 |  | - | |  | 0 | 1 |
| Fish and shellfish | | 0 | 1 |  | 0 | 1 |  | 0 | 1 |  | 0 | 1 |
| Seaweeds | | 0 | 1 |  | 0 | 1 |  | 0 | 1 |  | 0 | 1 |
| Green tea | | 0 | 1 |  | 0 | 1 |  | 0 | 1 |  | 0 | 1 |
| Beef and pork | | 1 | 0 |  | 1 | 0 |  | 1 | 0 |  | 1 | 0 |
| Coffee | | 1 | 0 |  | - | |  | - | |  | 1 | 0 |
| Fruit | | - | |  | - | |  | 0 | 2 |  | 0 | 1 |
| Soybeans and soybean foods | | - | |  | - | |  | 0 | 2 |  | 0 | 1 |
| Mushrooms | | - | |  | - | |  | 0 | 1 |  | 0 | 1 |
| Range of the score | | 0 to 9 | |  | 0 to 8 | |  | -1 to 12 | |  | 0 to 12 | |
| JDI, Japanese Diet Index; JDI8, 8-item Japanese Diet Index; wJDI9, 9-component-weighted Japanese Diet Index; mJDI12, 12-component modified Japanese Diet Index. | | | | | | | | | | | | |
| a | Zhang S, et al. A cross-sectional study of the associations between the traditional Japanese diet and nutrient intakes: the NILS-LSA project. Nutrition Journal. 2019;18. | | | | | | | | | | | |
| b | Tomata Y, et al. Dietary Patterns and Incident Dementia in Elderly Japanese: The Ohsaki Cohort 2006 Study. J Gerontol A Biol Sci Med Sci. 2016;71:1322-8. | | | | | | | | | | | |
| c | Lu Y, et al. Changes in a specific dietary pattern and incident dementia: A prospective cohort study. Clin Nutr. 2021;40:3495-502. | | | | | | | | | | | |

| **Supplementary Table 2**. HRs (and 95% CIs) for incident dementia by JDI8, JDI, and mJDI12 scores (all JDI scores were used as continuous and categorical variables) ^a^ | | | | | | | | |
| --- | --- | --- | --- | --- | --- | --- | --- | --- |
|  |  | No. of  participants | No. of  incident dementia (%) | Person  years | Multivariable adjusted model ^b^ | | | |
|  |  |  |  |  | HR | 95% CI | | *P*-value |
| Continuous JDI8 scores | | 1504 | 225 (15.0) | 16880.8 | 1.01 | 0.92 | 1.10 | 0.919 |
| Tertiles of JDI8 scores | |  |  |  |  |  |  |  |
|  | T1 | 587 | 74 (12.6) | 6521.9 | 1.00 | Ref. | | - |
|  | T2 | 316 | 55 (17.4) | 3497.0 | 1.45 | 1.01 | 2.09 | 0.044 |
|  | T3 | 601 | 96 (16.0) | 6861.9 | 1.07 | 0.78 | 1.47 | 0.688 |
|  |  |  |  |  |  |  |  |  |
| Continuous JDI scores | | 1504 | 225 (15.0) | 16880.8 | 1.00 | 0.92 | 1.09 | 0.975 |
| Tertiles of JDI scores | |  |  |  |  |  |  |  |
|  | T1 | 471 | 54 (11.5) | 5311.3 | 1.00 | Ref. | | - |
|  | T2 | 599 | 100 (16.7) | 6605.9 | 1.27 | 0.90 | 1.78 | 0.178 |
|  | T3 | 434 | 71 (16.4) | 4963.7 | 1.20 | 0.83 | 1.74 | 0.339 |
|  |  |  |  |  |  |  |  |  |
| Continuous mJDI12 scores | | 1504 | 225 (15.0) | 16880.8 | 0.96 | 0.90 | 1.03 | 0.287 |
| Tertiles of mJDI12 scores | |  |  |  |  |  |  |  |
|  | T1 | 373 | 43 (11.5) | 4106.1 | 1.00 | Ref. | | - |
|  | T2 | 525 | 89 (17.0) | 5746.3 | 1.04 | 0.71 | 1.52 | 0.838 |
|  | T3 | 606 | 93 (15.4) | 7028.4 | 0.88 | 0.59 | 1.30 | 0.520 |
| JDI8, 8-item Japanese Diet Index; JDI, Japanese Diet Index; mJDI12, 12-component modified Japanese Diet Index; HR, hazard ratio; CI, confidence interval. | | | | | | | | |
| a | Analysis by Cox proportional hazards model. | | | | | | | |
| b | Adjusted for baseline information on sex, APOE genotype (APOE-ε4 carriers: 2/4, 3/4, 4/4, or APOE-ε4 noncarriers: 2/2, 2/3, 3/3), body mass index (kg/m^2^; < 18.5, 18.5 – < 25, or ≥ 25), history of disease (stroke, hypertension, dyslipidemia, and diabetes mellitus; yes or no, for each), participation waves (categorical), smoking status (never, former, or current), alcohol intake (g/day; continuous), total physical activity (METs*hr/day; continuous), education level (years; ≤ 9, 10–12, or ≥ 13), depressive symptoms (CES-D score; ≤ 15 or ≥ 16), marital status (married, or others), energy intake (kcal/day; continuous), and MMSE score (continuous). | | | | | | | |

| **Supplementary Table 3**. HRs (and 95% CIs) for incident dementia by food components [g/day; daily intake < gender-specific median (ref.) vs. ≥ sex-specific median] ^a^ | | | | | | |  |
| --- | --- | --- | --- | --- | --- | --- | --- |
|  |  | Multivariable adjusted model ^b^ | | | | | |
|  |  | HR | 95% CI | | *P*-value | | |
| Rice | | 1.10 | 0.82 | 1.47 | | 0.537 | |
| Fish and shellfish | | 1.28 | 0.97 | 1.69 | | 0.077 | |
| Green and yellow vegetables | | 0.94 | 0.71 | 1.25 | | 0.688 | |
| Seaweeds | | 0.96 | 0.73 | 1.27 | | 0.792 | |
| Green tea | | 0.92 | 0.70 | 1.21 | | 0.561 | |
| Beef and pork | | 1.21 | 0.92 | 1.59 | | 0.176 | |
| Soybeans and soybean foods | | 0.72 | 0.54 | 0.95 | | 0.021 | |
| Mushrooms | | 1.01 | 0.77 | 1.32 | | 0.969 | |
| Fruit | | 0.85 | 0.63 | 1.14 | | 0.278 | |
| HR, hazard ratio; CI, confidence interval. | | | | | | |  |
| a | Analysis by Cox proportional hazards model. | | | | | |  |
| b | Adjusted for baseline information on sex, APOE genotype (APOE-ε4 carriers: 2/4, 3/4, 4/4, or APOE-ε4 noncarriers: 2/2, 2/3, 3/3), body mass index (kg/m^2^; < 18.5, 18.5 – < 25, or ≥ 25), history of disease (stroke, hypertension, dyslipidemia, and diabetes mellitus; yes or no, for each), participation waves (categorical), smoking status (never, former, or current), alcohol intake (g/day; continuous), total physical activity (METs*hr/day; continuous), education level (years; ≤ 9, 10−12, or ≥ 13), depressive symptoms (CES-D score; ≤ 15 or ≥ 16), marital status (married, or others), energy intake (kcal/day; continuous), MMSE score (continuous), and other food components simultaneously. | | | | | |  |

| **Supplementary Table 4**. HRs (and 95% CIs) for incident dementia by wJDI9 score [further adjusted for salt intake (g/day); wJDI9 scores were used as continuous and categorical variable] ^a^ | | | | | | | | | |
| --- | --- | --- | --- | --- | --- | --- | --- | --- | --- |
|  |  | No. of | No. of | Person | Multivariable adjusted model ^b^ | | | | |
|  |  | participants | incident dementia (%) | years | HR | 95% CI | | *P*-value | |
| Continuous wJDI9 scores | | 1504 | 225 (15.0) | 16880.8 | 0.95 | 0.90 | 1.00 | | 0.031 |
| Tertiles of wJDI9 scores | |  |  |  |  |  |  | |  |
|  | T1 | 549 | 94 (17.1) | 5992.8 | 1.00 | Ref. | | - | |
|  | T2 | 582 | 91 (15.6) | 6505.3 | 0.97 | 0.72 | 1.31 | | 0.833 |
|  | T3 | 373 | 40 (10.7) | 4382.7 | 0.58 | 0.40 | 0.86 | | 0.006 |
| wJDI9, 9-component-weighted Japanese Diet Index; HR, hazard ratio; CI, confidence interval. | | | | | | | | | |
| a | Analysis by Cox proportional hazards model. | | | | | | | | |
| b | Adjusted for baseline information on sex, APOE genotype (APOE-ε4 carriers: 2/4, 3/4, 4/4, or APOE-ε4 noncarriers: 2/2, 2/3, 3/3), body mass index (kg/m^2^; < 18.5, 18.5 – < 25, or ≥ 25), history of disease (stroke, hypertension, dyslipidemia, and diabetes mellitus; yes or no, for each), participation waves (categorical), smoking status (never, former, or current), alcohol intake (g/day; continuous), total physical activity (METs*hr/day; continuous), education level (years; ≤ 9, 10–12, or ≥ 13), depressive symptoms (CES-D score; ≤ 15 or ≥ 16), marital status (married, or others), energy intake (kcal/day; continuous), MMSE score (continuous), and salt intake (g/day; continuous). | | | | | | | | |
